# Supplementary material for: The Fabp4-Cre-Model is Insufficient to Study Hoxc9 Function in Adipose Tissue
Source: Biomedicines. 2020 Jun 29;8(7):184. doi: 10.3390/biomedicines8070184 (PMC7400597; doi:10.3390/biomedicines8070184)
Supplement: Supplementary file 1 [file biomedicines-08-00184-s001.pdf]

Article – Supplementary Material

Fabp4-Cre-mediated targeting of *Hoxc9* in adipose tissue

Sebastian Dommel[1*], Claudia Berger[1], Anne Kunath[1], Matthias Kern[1], Martin Gericke[2,3], Peter Kovacs[1], Esther Guiu-Jurado[1], Nora Klöting[1,4] and Matthias Blüher[1,4,*]

[1] Medical Department III – Endocrinology, Nephrology, Rheumatology, University of Leipzig Medical Center, Leipzig, Saxony, D-04103, Germany

[2] Institute of Anatomy, Leipzig University, Leipzig, Saxony, D-04103, Germany

[3] Institute of Anatomy and Cell Biology, Martin-Luther-University, Halle (Saale), Saxony-Anhalt, D-06108, Germany

[4] Helmholtz Institute for Metabolic, Obesity and Vascular Research (HI-MAG) of the Helmholtz Zentrum München at the University of Leipzig and University Hospital Leipzig, Leipzig University, Leipzig, Saxony, D-04103, Germany

* Correspondence: sebastian.dommel@medizin.uni-leipzig.de; Tel.: +49-341-9713400 (S.D.); matthias.blueher@medizin.uni-leipzig.de; Tel.: +49-341-9715984 (M.B.)

SUPPLEMENTARY DATA

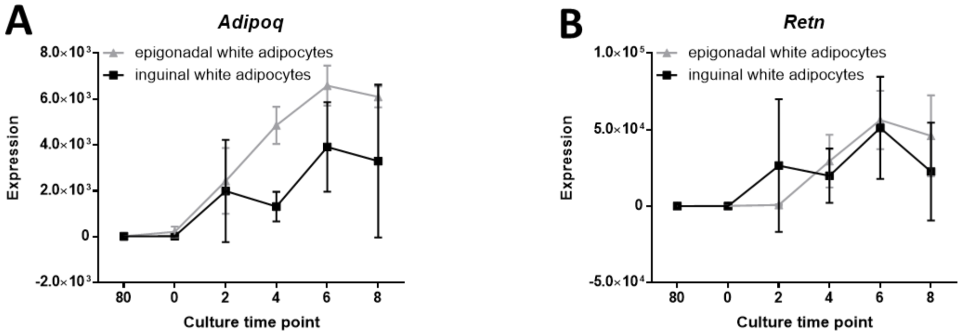

Figure S1: Gene expression patterns in immortalized white adipocytes of epigonadal or inguinal origin. (**A**) *Adipoq* expression during adipogenesis. (**B**) *Retn* expression during adipogenesis. Mapped time points were at 80% pre confluence (80), 100% confluence and initiation of differentiation (0) and days post confluence (2 - 8). Data presented as mean ± SD, N = 3.

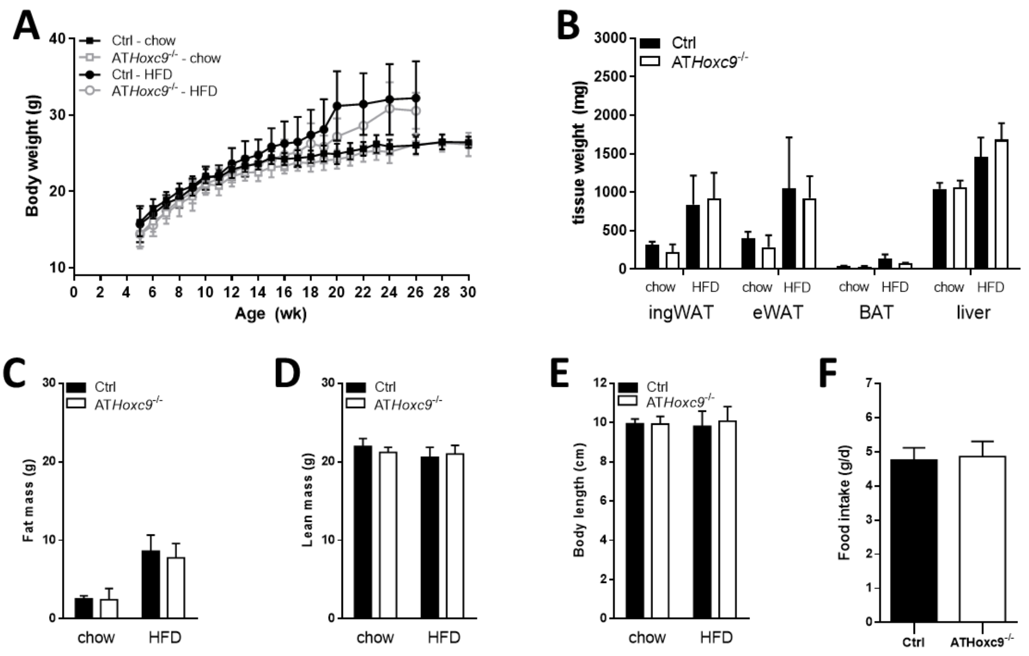

Figure S2: Phenotyping of female ATHoxc9 deficient mice. (A - F) Body weight gain, tissue weights, fat and lean mass as well as body length and food intake do not differ between ATHoxc9-/- and control female mice.

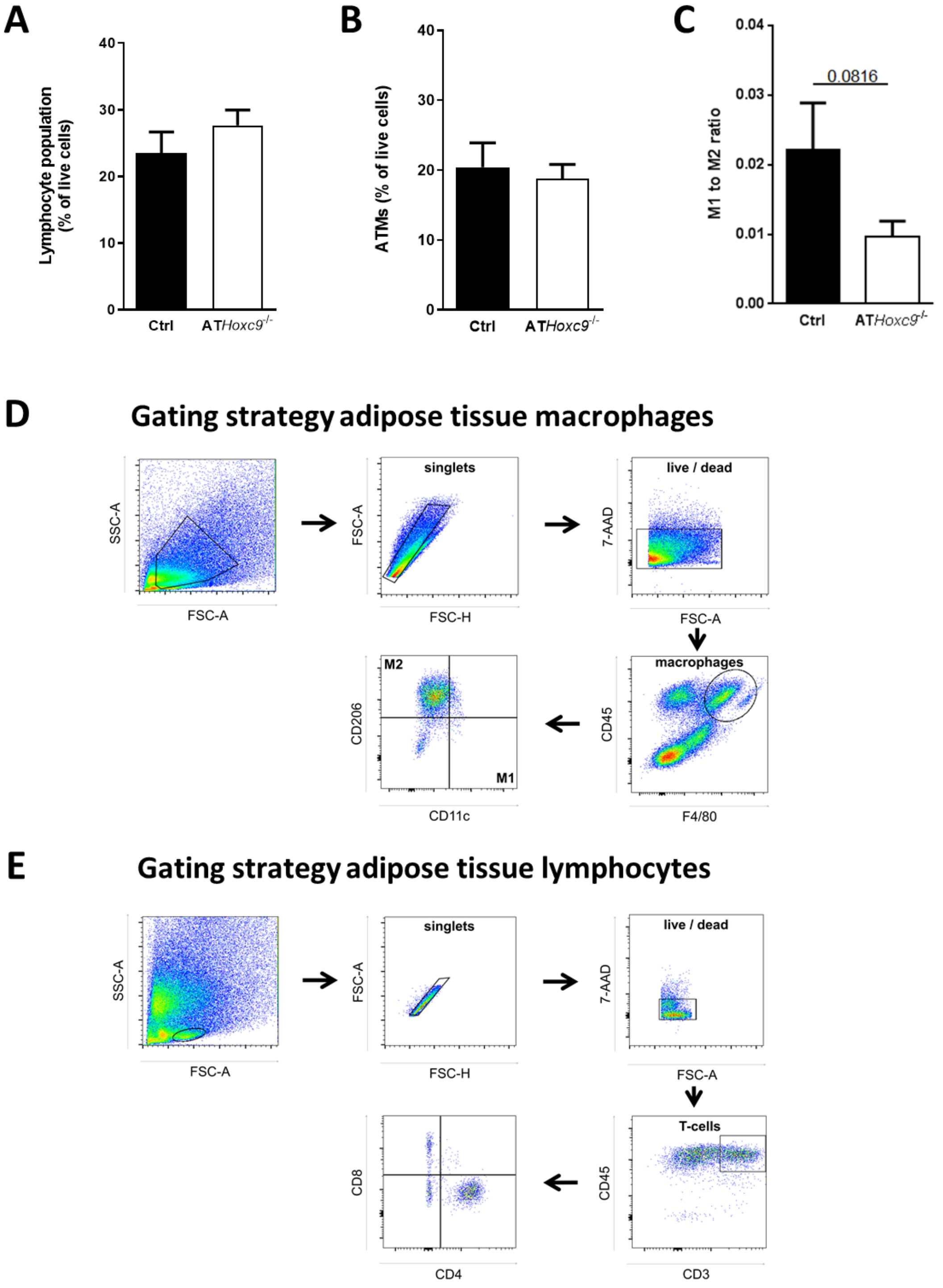

Figure S3: Consequences of *Hoxc9* targeting on adipose tissue immune cells. *(A - C)* Analysis of the immune phenotype show no differences in respect to lymphocyte and adipose tissue macrophage (ATM) populations. M1 to M2 macrophage ratio tend to be reduced in lean female AT*Hoxc9*$^{-/-}$ mice. *n* = 8 Ctrl vs. 10 AT*Hoxc9*$^{-/-}$ mice. Data represent mean ± SEM. *(D)* Representative flow cytometry gating strategy for living adipose tissue macrophages. *(E)* Representative flow cytometry gating strategy for living adipose tissue lymphocytes-

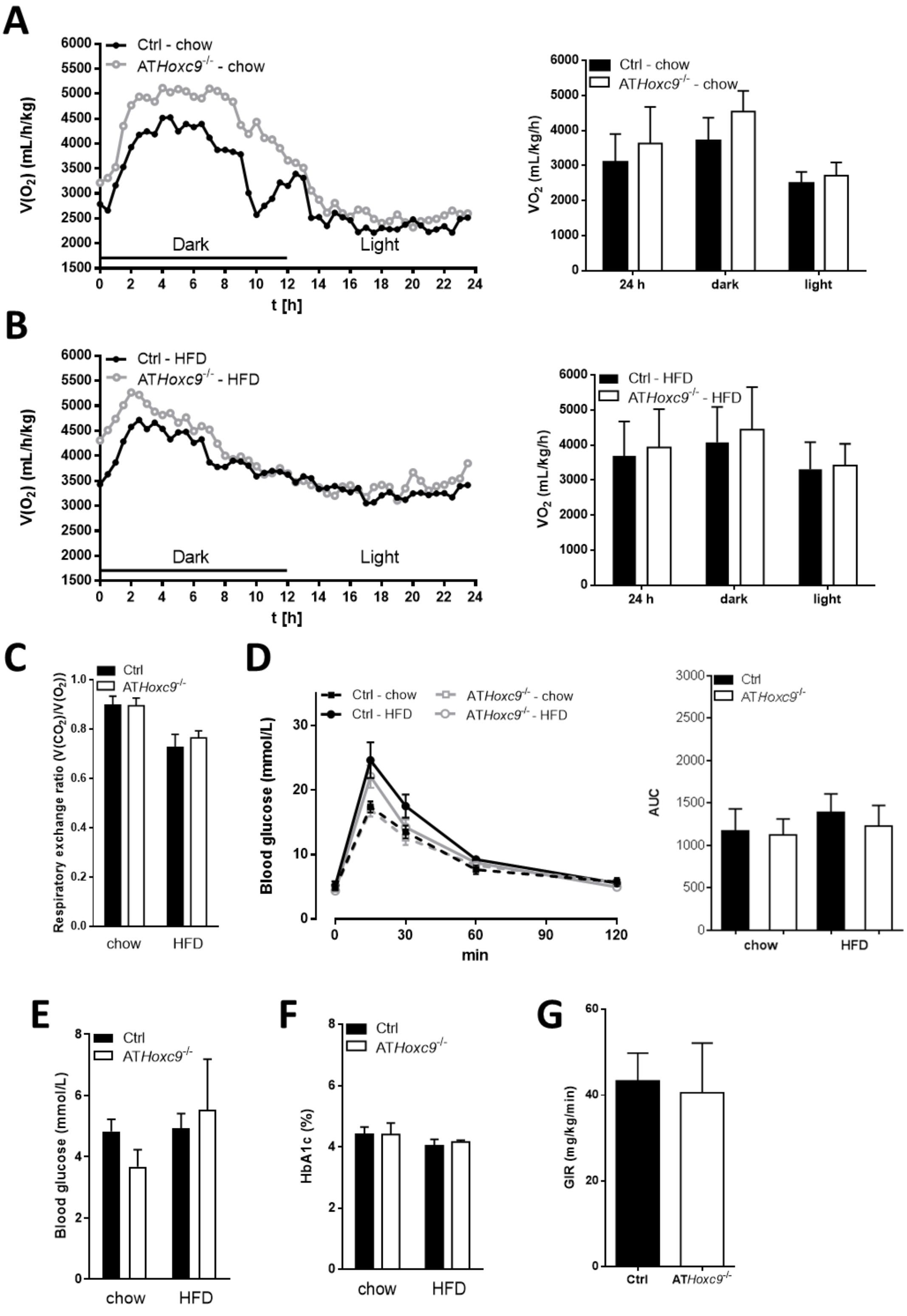

Figure S4: Metabolic parameters of AT*Hoxc9* deficient female mice. (A - C) Oxygen consumption and respiratory exchange rate (RER, C) was not altered by presence or absence of *Hoxc9* in AT neither in chow (A) nor in HFD fed mice (B). (D) Female AT*Hoxc9*$^{-/-}$ mice showed no difference in glucose tolerance in respect to diets compared to control animals during intraperitoneal glucose tolerance tests (GTT) after 25 weeks of age. (E) Fasting blood glucose and (F) long time HbA1c levels do not differ in female mice.

(G) Hyperinsulinemic-euglycemic clamps were performed in chow diet animals at 23 – 25 weeks of age to determine insulin sensitivity represented by glucose infusion rate (GIR,). *n*(A) = 3 Ctrl vs. 8 AT*Hoxc9*$^{-/-}$, *n*(B) = 6 Ctrl vs. 6 AT*Hoxc9*$^{-/-}$, *n*(D) = CD 8 Ctrl vs. 10 AT*Hoxc9*$^{-/-}$, HFD 7 Ctrl vs. 10 AT*Hoxc9*$^{-/-}$, *n*(E) = CD 8 Ctrl vs. 10 AT*Hoxc9*$^{-/-}$, HFD 9 Ctrl vs. 6 AT*Hoxc9*$^{-/-}$, *n*(F) = CD 4 Ctrl vs. 5 AT*Hoxc9*$^{-/-}$, HFD 9 Ctrl vs. 6 AT*Hoxc9*$^{-/-}$, *n*(G) = CD 4 Ctrl vs. 9 AT*Hoxc9*$^{-/-}$. Data represent mean ± SD.

Table S1. Primer pairs used for PCR and qPCR

Gene	forward (3' - 5')	reverse (3' - 5')	product size [bp]	
Hoxc9 - loxP site	CTCTGACTCTGAGACTACCCTTCC	GCATACAGCCTAGGTTTTCAGC	324 (lox) 205 (WT)	PCR
Hoxc9 - Intron 1 Exon 2	AAAAGCCACGTTCCGAACTG	GACGAGGTAGGTGGAGGAAC	115	PCR
Fabp4-Cre recombinase	GCGGTCTGGCAGTAAAAACTATC	GTGAAACAGCATTGCTGTCACTT	100	PCR
36b4	ACTGGTCTAGGACCCGAGAAG	TCAATGGTGCCTCTGGAGATT	77	qRT-PCR
Actb	GTGACGTTGACATCCGTAAAGA	GCCGGACTCATCGTACTCC	245	qRT-PCR
Adipoq	AAGGAGATGCAGGTCTTCTTGGT	CTGAACGCTGAGCGATACACAT	145	qRT-PCR
Fabp4	AAGGTGAAGAGCATCATAACCCT	TCACGCCTTTCATAACACATTCC	133	qRT-PCR
Hoxc9	ACTCGCTCATCTCTCACGACA	GGACGGAAAATCGCTACAGTC	119	qRT-PCR
Lep	TGAAGCCCAGGAATGAAGTC	TCAAGACCATTGTCACCAGG	97	qRT-PCR
